# Real benefit of a protective factor against dementia: Importance of controlling for death. Example of sport practice

**DOI:** 10.1371/journal.pone.0174950

**Published:** 2017-04-17

**Authors:** Leslie Grasset, Pierre Joly, Hélène Jacqmin-Gadda, Luc Letenneur, Jérôme Wittwer, Hélène Amieva, Catherine Helmer, Jean François Dartigues

**Affiliations:** 1 Univ. Bordeaux, Inserm, Bordeaux Population Health Research Center, UMR, Bordeaux, France; 2 Bordeaux University Hospital, Memory Consultation, CMRR, Bordeaux, France; Nathan S Kline Institute, UNITED STATES

## Abstract

**Objectives:**

To analyse the impact of a risk factor on several epidemiological indicators of death and dementia; the example of sport practice is presented.

**Methods:**

A population of 3670 non-demented subjects living at home and aged 65 and older from the PAQUID study were followed for 22 years. Sport practice was documented at baseline. Dementia (according to DSM-III-R criteria) and death were assessed at each visit. Analyses were performed with an Illness-Death model, providing results on the risks of dementia and death, probabilities and life expectancies.

**Results:**

A total of 743 subjects (20.2%) participated in regular sport practice. During the follow-up, the proportion of death was lower in the elderly people practicing sport (EPPS), whereas the proportion of incident dementia cases was the same. The adjusted model showed a decreased risk of dementia (HR = 0.84 (0.72–1.00)) and of death for non-demented subjects (HR = 0.61 (0.51–0.71)) for EPPS but a similar risk of death with dementia in both sport groups. The probability of remaining alive without dementia was higher in EPPS, whereas the probability of dying was lower. The mean lifetime without dementia was 3 years higher for the EPPS, but the mean lifetime with dementia was the same.

**Discussion:**

A preventive measure on a protective factor that is more effective for preventing death than dementia could lead to an increased lifetime without dementia; however, the number of demented cases may remain unchanged, even if the risk of developing dementia is reduced. This dynamic is important to forecast the need for health care and social services for the elderly.

## Introduction

Dementia is a major cause of disability in the elderly and is a major fear in this period of life [1]. Unfortunately, despite enormous research effort, no progress has been made in the curative treatment of dementia for approximately twenty years. Hopefully, some epidemiological studies showing a decrease in incidence and prevalence of dementia are in favour of possible successful prevention strategies [2–7]. Among the candidate factors for prevention, regular physical activity is one of the most promising [8–14]. However, the important question from an individual and collective point of view is: what results can we expect if we enhance regular physical activity and sport in the elderly?

Indeed, beyond a possible decrease in the risk of dementia, the real benefit, from an individual point of view, is to increase the duration of life without dementia [15–17] and, from a collective point of view, to decrease the number of demented patients in the general population. Regular physical activity is not only a protective factor against dementia but also a protective factor against death, although beneficial effect of regular physical activity can also partly come from other cognitive or socially active lifestyle, often associated. We recently showed that a factor reducing the risk of death and, to a lesser extent, the risk of dementia could lead to increased survival of frail subjects and thus increase the number of people likely to develop dementia [18]. The competition between both events (death and dementia) means that beyond an apparent benefit with respect to the risk of disease, the benefits with respect to the prevalence (in this paper we considered prevalence to be the number of cases and not the rate) of dementia is not clear.

The aim of this paper is to analyse the real impact of a protective factor (i.e., sport practice) on the risks of death and dementia in elderly French people, evaluating its effects on the probability of developing dementia and on the duration of life before and after dementia using an appropriate illness-death model applied to the PAQUID cohort study.

## Methods

### Sample selection and follow-up of the cohort

The Personnes Agées QUID (PAQUID) study, a French prospective cohort study, aims to study cognitive ageing and loss of autonomy. The cohort was randomly selected from electoral rolls and included 3777 subjects aged 65 years or older at baseline who were living at home in two administrative areas of southwestern France (Gironde and Dordogne). Subjects were visited at home by a trained psychologist at baseline in 1988/1989 and then again approximately 1, 3, 5, 8, 10, 13, 15, 17, 20 and 22 years after the initial visit. Full details of the study have been described elsewhere [19]. An ethical review committee approved the PAQUID study.

### Data collection

At each visit, a questionnaire was administered at home by a neuropsychologist, including information about sociodemographic characteristics, lifestyle and health characteristics, medication consumption, a battery of cognitive tests, and scales of disability. Vital status was systematically recorded for all participants. Dementia was assessed at each visit using a two-stage procedure: subjects who met the Diagnostic and Statistical Manual of Mental Disorders, Third Edition, Revised (DSM-III-R) criteria for dementia, as assessed by the neuropsychologist, underwent clinical assessment by a neurologist who ascertained the final diagnosis. All cases were reviewed by a group of experts.

Leisure and social activities were documented at baseline by a standardised questionnaire during the face-to-face interview conducted by the psychologist. Ten activities were recorded with the question: “Do you usually undertake this activity (at least once a week): yes or no?” The following activities were screened: reading, gardening, doing odd jobs or knitting, watching television, participating in sports, playing board games, looking after children, participating in group activities or associations, visiting friends or family members and travelling. Only sport practice was considered in this paper. Age at baseline, gender, educational level, initial cognitive level based on the Mini-Mental State Examination (MMSE), stroke history, diabetes, and antihypertensive drug use were also documented by the baseline questionnaire. The PAQUID study protocol was approved by an ethic committee, and all participants gave their informed consent.

### Statistical analysis

The sociodemographic characteristics and health events were first described and compared according to sport practice status using chi-squared test and Student’s t-test. Then, analyses were performed using a semi-parametric illness-death model accounting for both competing mortality and interval censoring, i.e., the possibility of developing dementia between the last dementia-free visit and death [20]. This model links three states: alive without dementia (state 0), alive with dementia (state 1) and dead (state 2). The transition intensities between each state were estimated; these transition intensities can be interpreted as incidences: incidence of dementia (transition from state 0 to state 1); incidence of death for people without dementia (transition from state 0 to state 2); and incidence of death for people with dementia (transition from state 1 to state 2). Subjects lost to follow-up were censored for dementia at their last visit. Using the parameter estimates of this illness-death model, the following predictive parameters were computed: 1) the probability of being alive without dementia according to the duration of follow-up; 2) the probability of being alive with dementia; 3) the probability of dying; 4) the mean remaining lifetime with and without dementia. Each parameter is presented for subgroups of sport practice. Adjusted hazard ratios are presented and the probabilities and life expectancies are given for different types of individuals, allowing for determination of mean life expectancies.

We analysed the risk of developing dementia in three different models: 1) adjusted for sport practice and age; 2) additionally adjusted for gender, education (at least primary school certificate versus no diploma) and MMSE score at baseline; and 3) additionally adjusted for stroke (self-reported), diabetes (self-reported and/or antidiabetic treatment) and antihypertensive treatment. The exponentials of the regression coefficients can be interpreted as a hazard ratio (HR) and were simultaneously estimated using a penalised likelihood implemented in the SmoothHazard R package [20].

## Results

Among the 3777 participants, 3670 were non-demented at baseline and provided answers to the leisure and social activities questionnaire (5 were missing values for sport practice status). Among them, 743 participated in regular sport practice (20.2%). The elderly people practicing sport (EPPS) were younger and had a higher level of education and better cognitive performance at baseline than the others (Table 1). The proportion of EPPS was higher in men than in women. During the 22 years of follow-up, 577 subjects (77.7%) from the EPPS group died and 168 were diagnosed with dementia (22.6%), whereas among those practicing no sport, 2556 died (87.3%) and 691 were diagnosed with dementia (23.7%). The mean MMSE score at time of diagnosis of dementia was 18.4 (5.4). The mean follow-up time was 11.57 (6.9) years.

**Table 1:** Characteristics of the population according to sport practice status, n = 3670.

	No sport practice	Sport practice	P value
	N = 2927	N = 743	
Gender Women	1809 (61.8)	320 (43.1)	<0.0001
Age at baseline			<0.0001
65–75	1398 (47.8)	481 (64.8)	
75–85	1188 (40.6)	232 (31.2)	
85 +	341 (11.6)	30 (4.0)	
Mean (SD)	75.78 (6.96)	73.24 (5.73)	<0.0001
Education			<0.0001
No diploma	1082 (37.0)	195 (26.2)	
At least primary school certificate	1845 (63.0)	548 (73.8)	
Baseline MMSE performance*: mean (SD)	25.57 (3.54)	26.69 (2.85)	<0.0001
History of stroke	178 (6.1)	16 (2.2)	<0.0001
Diabetes	265 (9.1)	41 (5.5)	0.002
Anti-hypertensive drug use	1654 (56.5)	346 (46.6)	<0.0001

Unless otherwise indicated, data are expressed as n (%)

^*^ 75 missing values

Using the illness death model with sport practice adjusted for age, the instantaneous risk of dementia was significantly decreased in EPPS (HR = 0.79, 95% Confidence Interval (95% CI): 0.67, 0.93) (Table 2, model 1). The risk of dying was also decreased in non-demented EPPS, with a higher magnitude than the risk of dementia (HR = 0.67, 95%CI: 0.57, 0.79), whereas the risk of dying was unchanged in demented EPPS (HR = 1.06, 95%CI: 0.90, 1.26, *p* = 0.47).

**Table 2:** Estimated hazard ratio for sport practice on dementia incidence and mortality of non-demented and demented subject.

	Model 1			Model 2			Model 3		
	(n = 3670)			(n = 3595)			(n = 3595)		
	HR	(95% CI)	p value	HR	(95% CI)	p value	HR	(95% CI)	p value
**Incidence of dementia**									
Sport practice	0.79	0.67, 0.93	0.006	0.85	0.72, 1.00	0.046	0.84	0.72, 1.00	0.044
**Mortality without dementia**									
Sport practice	0.67	0.57, 0.79	<0.0001	0.59	0.50, 0.69	<0.0001	0.61	0.51, 0.71	<0.0001
**Mortality with dementia**									
Sport practice	1.06	0.90, 1.26	0.47	0.96	0.81, 1.13	0.61	0.97	0.82, 1.15	0.73

HR: Hazard Ratio; CI: Confidence Interval

Model 1: Adjusted for age

Model 2: Adjusted for age, gender, education and baseline MMSE score (75 missing values)

Model 3: Adjusted for age, gender, education, baseline MMSE score (75 missing values), stroke, diabetes, anti-hypertensive drug use

After adjustment for gender, education and MMSE score at baseline, the hazard ratios for death associated with sport practice remained almost unchanged, but the HR for dementia was slightly increased (HR = 0.85, 95%CI: 0.72, 1.00, *p* = 0.046) (Table 2, model 2). After additional adjustment for stroke, diabetes and antihypertensive drug use, the HR for dementia in EPPS remained at 0.84 (95%CI: 0.72, 1.00, *p* = 0.044), and the HR for death in non-demented and demented subjects remained unchanged.

**Fig 1:**
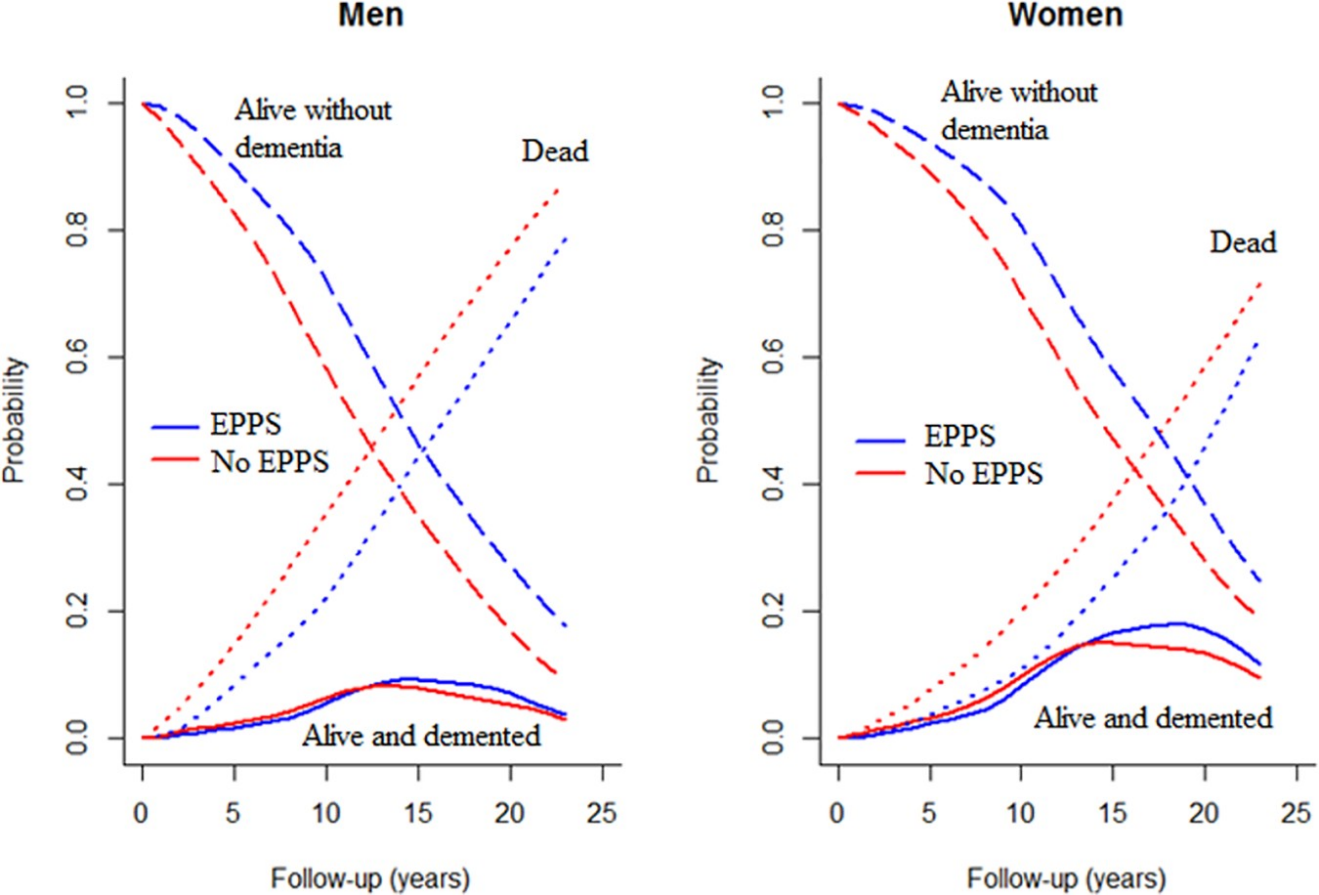
Probabilities of being in a given state (alive without dementia, dead, alive and demented) according to sport practice status and year of follow-up since measure of sport practice (EPPS: Elderly People Practicing Sport).

The probabilities of being in one of the three states (demented, non-demented, and dead) according to the duration of follow-up (FU) of the cohort are presented in Fig 1 for two individuals: a man and a woman aged 70 at baseline with a diploma. Whatever time in the 20 years following the measure of sport practice and compared to non-EPPS, the probability of being alive and non-demented was higher for EPPS, and the probability of dying was lower. The probability of being alive with dementia was almost the same in EPPS and non-EPPS regardless of the duration of follow-up, but the probability was higher for a woman aged 70 with a diploma than for a man with the same characteristics.

The mean lifetimes without and with dementia are presented in Table 3 and in Web Table 1, respectively, according to age, sex and diploma. Whatever age, sex, and diploma, the mean lifetime without dementia was higher in EPPS, whereas there was no difference in the mean lifetime with dementia according to EPPS. For example, in EPPS, the mean lifetime without dementia for a man aged 70 with a diploma was 14.42 years (95%CI: 12.93, 14.92), more than two years longer than in a similar non-EPPS man (12.22 years, 95%CI: 11.67, 12.64). In contrast, the mean lifetime with dementia was almost the same in both groups (3.26 vs 3.81 years) (S1 Table). For a woman aged 70 with a diploma, the mean lifetime without dementia for EPPS was 16.22 years (14.51, 16.67), whereas it was 14.45 years (13.89, 14.77) for a similar non-EPPS woman. Again, the lifetime with dementia was the same in both groups (4.32 vs 4.82 years) (S1 Table).

**Table 3:** Mean lifetime without dementia according to age, sex, diploma, and sport practice status.

		Men				Women			
		Without diploma		With diploma		Without diploma		With diploma	
		Year	CI	Year	CI	Year	CI	Year	CI
**70 years**	**EPPS**	13.29	11.59, 14.08	14.42	12.93, 14.92	14.99	13.04, 15.61	16.22	14.51, 16.67
	**No EPPS**	10.73	10.05, 11.24	12.22	11.67, 12.64	12.49	11.83, 12.92	14.45	13.89, 14.77
**80 years**	**EPPS**	8.01	5.89, 8.50	8.91	6.78, 9.36	9.44	6.58, 9.92	10.54	7.57, 11.08
	**No EPPS**	5.84	5.41, 6.14	6.83	6.43, 7.09	7.11	6.54, 7.35	8.59	8.09, 8.86
**85 years**	**EPPS**	6.07	3.54, 6.53	6.78	4.33, 7.24	7.27	3.84, 7.73	8.15	4.71, 8.71
	**No EPPS**	4.17	3.80, 4.42	4.91	4.55, 5.17	5.20	4.65, 5.41	6.34	5.84, 6.59

CI: Confidence Interval

EPPS: Elderly People Practicing Sport

Taking into account the age, sex, and diploma distributions in the PAQUID sample, the mean increase of lifetime without dementia for Elderly People Practicing Sport was 3.19 years compared to those who do not practice sport (13.38 vs 10.19 years).

## Discussion

With an illness-death model applied to the long-term follow-up of the PAQUID cohort, we found a higher probability of remaining alive without dementia in EPPS with a mean increase of 3.19 years of the mean lifetime without dementia in elderly subjects participating in sport compared to those not participating in sport. These 3.19 years represent an increase of almost 31.3% of the mean lifetime without dementia, which is substantial and of interest from an individual point of view. Estimations of “dementia-free life expectancy” have already been provided on the basis of prevalence data from the PAQUID cohort [21], which have been replicated in several countries [22–24]; however, the statistical analysis used did not allow study of the relationships with multiple risk factors or predictors of death and dementia.

However, although the incidence of dementia was decreased in EPPS by more than 20% compared to non-EPPS, the probability of developing dementia over the 22-year follow-up period was higher for EPPS than for non-EPPS. This finding can be explained by the fact that the protective effect of practicing sport is far higher for the risk of dying than for the risk of developing dementia (0.61 vs 0.84), with a higher probability of dying for non-EPPS and thus a higher number of surviving EPPS at risk of developing dementia at later ages. From a collective point of view, a protective factor with a decreased risk of dementia can be of limited benefit to control the number of demented patients in the general population.

Similar results were found by Jacqmin-Gadda et al. with a simulated intervention on high blood pressure [18]. They showed that reducing the prevalence of high blood pressure on the whole population would lead to a decrease in both dementia incidence rates and mortality but would have a modest impact on the number of dementia cases. Our findings on the impact of sport practice support these theoretical simulations and underline the necessity to control for a semi-competing event, such as death, to evaluate the benefit of prevention strategies on the burden of dementia.

Contrary to the HR for dementia and mortality without dementia, sport practice had no effect on mortality risk among demented. Yet, demented sport participants had better cognitive and functional status at the time of dementia diagnosis than demented but non-sport participants (data not shown). We may assume that, despite the lack of benefit for mortality, being involved in sport could improve functional and social status, and thus quality of life in people living with dementia.

A weakness of our study is the assessment of sport practice by a single question regarding the regular practice of sport during the week preceding the interview. Assessments of regular midlife practice would have been more sensitive to evaluate the real impact of sport. In addition, although risks were adjusted for several potential risk factors, residual confounding cannot be excluded. However, the objective of this paper was to document the potential impact of risk factors on several epidemiological indicators and not to accurately evaluate the impact of sport. Indeed, we cannot conclude from these results that sport practice has a causal role in the increase in mean lifetime without dementia. Our study is observational, and even though we confirmed previous findings, [25] the evidence is not sufficient. Moreover, several randomised clinical trials have failed to find a benefit of recent sport practice on cognition.[26] Thus, our results showing a longer mean lifetime without dementia for EPPS compared to those not doing sport could be due to the protective role of a history of sport practice. The results could also be caused by sport practice being a marker of general healthy status, which could explain the lower risk of dementia. Indeed, sport practice is a marker of socially active lifestyle and part of its effect on dementia can also come from social factors, often associated, such as being part of a club or association, visiting friends or family, … However, this paper mainly investigated the impact of a protective factor on prevalence, incidence and life expectancies, with sport practice only being an illustration.

The strengths of this study are the prospective design of a large population-based cohort with a follow-up over more than twenty years and a careful collection of incident cases of death and dementia. We used an appropriate statistical model to model both the risk of dementia and death, taking into account interval censoring; moreover, we adjusted for several risk factors. This model provides better estimates and is more accurate than a standard survival model when interval censoring occurs [27]. The model is easily implemented using the R package SmoothHazard.

In conclusion, by controlling for the risk of death, the illness-death model provides useful original information on the real impact of potential prevention factors regarding the risk of dementia and the life expectancy of people without dementia. Each of these epidemiological indicators is of great value. From a public health point of view, the efficacy of an intervention designed to decrease the risk of dementia in the general population may have no impact on the overall number of demented subjects if the effect of the targeting protective factor is stronger against death than against dementia. However, extending the duration of life without dementia by applying preventive strategies is an obvious individual benefit.

## Supporting information

S1 Table Mean lifetime with dementia according to age, sex, diploma, and sport practice status. (DOCX)
