# Supplementary material for: Real benefit of a protective factor against dementia: Importance of controlling for death. Example of sport practice
Source: PLoS One. 2017 Apr 17;12(4):e0174950. doi: 10.1371/journal.pone.0174950 (PMC5393553; doi:10.1371/journal.pone.0174950)
Supplement: S1 Table — (DOCX) [file pone.0174950.s001.docx]

| Web Table 1: Mean lifetime with dementia according to age, sex, diploma, and sport practice status. | | | | | | | | | |
| --- | --- | --- | --- | --- | --- | --- | --- | --- | --- |
|  |  | **Men** | | | | **Women** | | | |
|  |  | **Without diploma** | | **With diploma** | | **Without diploma** | | **With diploma** | |
|  |  | Year | CI | Year | CI | Year | CI | Year | CI |
| 70 years | **EPPS** | 4.58 | 3.83, 6.58 | 3.26 | 2.66, 4.39 | 5.67 | 4.95, 7.72 | 4.32 | 3.70, 5.85 |
|  | **No EPPS** | 4.51 | 3.94, 5.22 | 3.81 | 3.33, 4.35 | 5.67 | 5.19, 6.38 | 4.82 | 4.39, 5.33 |
| 80 years | **EPPS** | 3.15 | 2.11, 4.64 | 2.03 | 1.39, 2.99 | 4.53 | 3.59, 6.32 | 3.02 | 2.45, 4.29 |
|  | **No EPPS** | 2.80 | 2.34, 3.25 | 2.32 | 2.01, 2.67 | 3.90 | 3.56, 4.39 | 3.20 | 2.94, 3.53 |
| 85 years | **EPPS** | 2.30 | 1.28, 3.83 | 1.47 | 0.77, 2.53 | 3.53 | 2.24, 5.40 | 2.30 | 1.56, 3.58 |
|  | **No EPPS** | 2.04 | 1.65, 2.46 | 1.71 | 1.42, 2.08 | 2.96 | 2.56, 3.45 | 2.44 | 2.19, 2.77 |
| CI: Confidence Interval  EPPS: Elderly People Practicing Sport | | | | | | | | | |
